# Breastfeeding and transmission of cytomegalovirus to preterm infants. *Case report and kinetic of CMV-DNA in breast milk*

**DOI:** 10.1186/1824-7288-37-6

**Published:** 2011-01-19

**Authors:** Manuela Chiavarini, Patrizia Bragetti, Alessandra Sensini, Elio Cenci, Roberto Castronari, Marta J Rossi, Ambra Fantauzzi, Liliana Minelli

**Affiliations:** 1 Department of Medical and Surgical Specialities and Public Health, Public Health section, University of Perugia School of Medicine, Via del Giochetto, 06125 PG, Italy; 2 Neonatal Intensive Care Unit, Teaching Hospital of Perugia, Italy; 3 Department of Microbiology, University of Perugia School of Medicine, Italy

## Abstract

**Background:**

Breastfeeding has a major impact on CMV epidemiology. Postnatal CMV reactivation's incidence during lactation is nearby the maternal seroprevalence. Although perinatal CMV infection has practically no consequences in term newborn, it may cause, in some cases, a severe symptomatic disease in preterm newborns.

The aims of the present study are to evaluate the rate and clinical expression of CMV infection breast milk transmitted in preterm infants and to check the safety of the freezing treated breast milk.

**Methods:**

The study included fifty-seven preterm infants and their CMV seropositive mothers. Fresh breast milk samples have been collected from 1^st ^to 9^th ^postpartum week. Both fresh breast milk and 72, 96, 120 hours frozen samples have been examined, checking the presence of CMV; urine samples have been tested too.

**Results:**

70.2% of tested mothers showed reactivation of the infection, and CMV-positive breast milk during the six weeks postpartum has been found. However, only one infant was infected by CMV, developing hepatic affection concomitantly with a multi-system involvement, as shown CMV DNA detection in urine, saliva, blood, gastric aspirate, and stools.

**Conclusion:**

Freezing breast milk at -20°C and pasteurization may respectively reduce or eliminate the viral load.

## Background

Human breast milk is considered as an ideal food for newborns, both for term and preterm infants, because of its nutritional value, unique qualities and properties. However it can also be a vehicle for viral and bacterial infections, as the breastfeeding is known to have a major impact on the epidemiology of postnatal cytomegalovirus (CMV) infection [1]. Because of an unknown mechanism, in women who are positive for anti-CMV IgG antibodies during breastfeeding viral reactivation can occur in the mammary glands and CMV may be generally excreted in the milk without clinical or laboratory signs of systemic infection (negative serum IgM, negative viruria) [2]. In premature and/or low birth weight (LBW) infants, however, serious consequences may be brought by breast milk-acquired CMV infection. At the moment, premature infants who are at risk for being infected by CMV from breast milk are not guideline-filed to be identified [3,4].

The aim of the study is to evaluate the rate and clinical expression of CMV infection transmitted by breast milk in preterm infants, born before 32 gestational weeks and/or weighting ≤2000 g at birth, checking the safety of the freezing treated breast milk.

## Methods

### Patients

The study has been carried out by Neonatology Intensive Care Unit (NICU) of the Teaching Hospital, in Perugia (Italy) from 1^st ^January 2004 to 31^st ^December 2007. Fifty seven preterm infants and their mothers, who were found CMV seropositive, were included in the prospective study. The study have been included preterm infants aged of < 32 complete weeks, or weighting < 2000 g at birth. Since the second life day. each newborn has been fed by naso-gastric tube (NGT).

Peripheral blood sample has been taken from mothers after delivery, testing CMV antibodies, to determine maternal serology. CMV immunoglobulin IgG and IgM antibodies have been determined, using enzyme immunoassay kits (CMV IgG: Diamedix Miami Florida USA, CMV IgM: Diasorin Saluggia Italy).

### Microbiology

Fresh breast milk samples have been collected from the 1th to 9th postpartum week. Fresh breast milk and 72, 96, 120 hours frozen samples have been tested, checking the presence of CMV. Milk samples have been filed and separated into cellular and aqueous fractions by centrifugation for obtaining. viral isolation. CMV culture has been undertaken on aqueous fraction, using the rapid shell vial tissue culture on MRC-5 cells (Vircell, Santa Fé, Spain). After two days incubation at 37°C, the cultures have been tested checking the presence of immediate-early IE1 and IE2 (MW 68-72 kDa) CMV antigens by direct immunofluorescence, using FITC-labelled monoclonal antibodies (Chemicon Light Diagnostics, Temecula, CA, USA). Milk samples DNA have been extracted by easyMAG (bioMérieux, Marcy l'Etoile, France) as molecular tests Nested PCR has been used as screening test, and quantitative real time PCR (RT-PCR) has been used to quantify the viral DNA load (Nanogen Advanced Diagnostics s.r.l., Buttigliera Alta, Torino, Italy). All samples have been assayed one by one. A negative control (distilled water) and a positive control (a DNA construct from CMV genome) have been included in each nested PCR. The lowest limit of detection was 10 copies/5 μl and 105 genomes/ml, for nested PCR and RT-PCR, respectively. Milk has been collected together with urine samples, to determine the viruria through quantitative and qualitative methods. Each infant has been fed by thawed maternal 72 hours frozen breast milk. Clinical status and laboratory tests have been documented during the study time. By informed consent, the parents of participating infants agreed them to be included in this study.

## Results

### Milk samples

**Figure 1:**
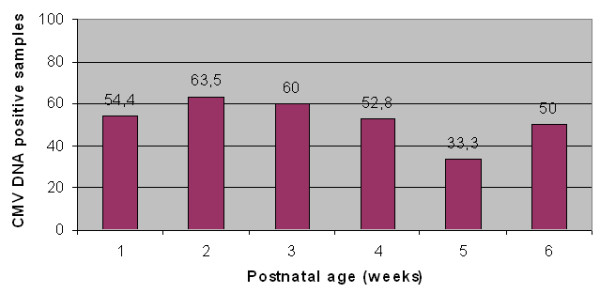
**CMV DNA kinetics**.

Previous viral infected mothers have been identified by IgG seropositivity towards CMV, in absence of IgM. In 40 out of 57 seropositive mothers (70.2%) the virus reactivated and was excreted in the milk during the six weeks postpartum lactation period. Maternal CMV reactivation was shown by viral shedding in breast milk (Figure 1) concomitantly with positive serum IgG but negative IgM, and negative viruria.

**Figure 2:**
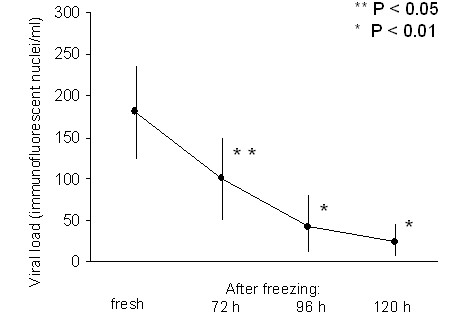
**Load virus in milk at different time**.

There was not statistically significant difference (p = 0.36) in the values of CMV kinetics, comparing positive samples to total samples examined in the different weeks. The majority of the colostrum samples were CMV DNA positive (31 out of 57, 54.4%) and most milk samples became CMV DNA positive two weeks after delivery (33 out of 52, 63.5%). During the period of the study 109 samples were tested and the highest values of CMV DNA copies, ranging between 10^4 ^to 10^6 ^copies/mL, were shown from the 4th to the 6th week after delivery. In this period the average DNA load was 61958,8 ± 15818,3 copies/mL. Thereafter, DNA copy number decreased progressively. The 72 h freezing process was capable of decreasing significantly the infectivity, as shown by viral culture, and the viral load (by approximately 75% (Figure 2). However, positive samples remained positive when tested by PCR, since viral DNA is detectable, even in the presence of very few inactivated CMV particles.

### Infants

The average gestational age of the newborns was 29 weeks (range 23-34 weeks). The average birth weight was 1158 ± 436.3 g. Among the infants who have been fed with CMV DNA-positive breast milk, only one became infected with CMV. Considering all the checked women, the rate of postnatal infection in exposed newborns was 2.5%: the rate of perinatal infection was 1.7%. The postnatal rate infection (2.5%) has been considered, because the infant became CMV positive 8 weeks after birth and the mother's serological test did not change, showing CMV IgM negative and IgG positive. This results indicate that maternal CMV reactivation was not systemic but occurred only in the breast.

All the infants were followed up to detect long-term sequele as described for congenital and perinatal cytomegalovirus infection: each infant underwent serial monitoring (on 3^rd^, on 6^th^, on 12^th^, on 24^th^, on 36^th ^month and on 5^th ^of correct gestation age). To date (after at least 3 years from the enrolment), none of them presents health problems correlated with CMV infection, including the symptomatic infants.

### Case report

The infant infected by CMV was born by caesarean section, carried out in election because of pre-eclampsia and intrauterine growth retardation, at 28th week and 3 days, having a birth weight of 740 g. He was initially treated for respiratory distress syndrome and hyperbilirubinaemia. During the first day of life, a wide spectrum antibiotic therapy as well as Immunoglobulin IgM-enriched have been administered, because of the increasing value of C-reactive protein in absence of other clinical symptoms. However, all tests performed to detect bacterial infectious were negative: gastric aspiration and tracheobronchial aspiration, including culture test for Mycoplasma and Clamidia, and blood culture. At age 30 days, the newborn developed conjugated hyperbilirubinaemia, and the liver ultrasound showed contracted gallbladder in accordance with relevant parietal thickening. On day 47, the clinical condition of the newborn worsened: elevated serum C-reactive protein levels were found and the infant was again treated with multiple antibiotics because of a suspected sepsis, even if blood and urine cultures were negative. Cholestasis indexes worsened (Bilirubin total 14,99 mg/dL, Bilirubin conjugated 8,31 mg/dL, AST 493 U/L, ALT 183 U/L, GGT 98 U/L). Moreover, the presence of CMV DNA was evidenced in urine, saliva and blood, and of CMV-specific IgM antibodies were positive in serum. The involvement of the digestive system by CMV was confirmed by the presence of a high number of viral DNA copies detected by PCR in the gastric aspirate and stools. Congenital transmission was excluded by negative CMV DNA detection from umbilical cord and Guthrie card, even using highly sensitive real-time PCR. Clinical improvement occurred in accordance with intravenous ganciclovir therapy (*7,5 mg/Kg/die twice/day for five days) *followed by administration of *valganciclovir (25 mg/Kg/die for six months)*[5]. The viral load gradually decreased until negativization. Molecular profiles of CMV strain isolated from infants' urine were indistinguishable from that isolated from maternal breast milk, indicating vertical CMV transmission through breast milk.

## Discussion

Sepsis- like syndrome in premature infants can be caused by acquired postnatal CMV infection, as reported by several studies [4], [6-8]. In order to elucidate the role of breastfeeding in transmission of CMV infection, this study has analysed prospectively both maternal CMV reactivation during lactation and the clinical outcome of primary infection of breastfed preterm infants. In breast milk whey CMV DNA is detectable with more reliability than in unfractionated milk or milk cells [9-12]. A very high proportion of CMV reactivation in longitudinally screened seropositive mothers during lactation has been found by RT-PCR, as viral DNA has been detected in breast milk from 40 out of 57 seropositive mothers (70.17%).

The mechanism of CMV reactivation in human milk, and the role of milk cells and cell-free virus in vertical transmission are still unknown [10]. Maternal risk factors for CMV transmission are considered to be early excretion of viral DNA and infectious virus in milk whey. The duration of breastfeeding has been related to the acquisition of CMV infection by term infants. Viral load in breast milk has not been correlated to CMV transmission. Kinetic PCR analysis performed on breast milk indicated that some samples collected during the first week after delivery were negative to CMV; viral DNA became detectable in most of the samples in the third week, but the copy number increased in the total samples examined from the 4th to the 6th week and decreased thereafter. Since the incubation time of CMV infection is between 30 and 120 days, mother-to-infant infections transmitted via breast milk should not occur until at least 6 weeks after delivery. However, more detailed studies are needed to elucidate the kinetics of CMV reactivation in the milk to reveal clearly risk factors for transmission. In only one case CMV DNA was detected in the urine 8 weeks after delivery. The newborn was not deliveried vaginally, therefore he might not have acquired CMV infection from vaginal secretions during the birth process. Hamprecht et al. have reported that the transmission of CMV from breastfeeding mothers to their preterm infants could result in symptomatic CMV infections, such as sepsis-like disease, and that the early onset of symptomatic infections occurs only in extremely immature preterm infants [4,12].

In a previous study, all term infants shed CMV into urine over a long period, and all of them had normal clinical courses without sequelae, but two preterm infants developed pneumonia [13]. Without giving sufficient evidence for breast milk as the maternal source of postnatal virus transmission, other reports have described symptomatic cytomegalovirus infection of preterm infants with clinical symptoms including neutropenia, thrombocytopenia, hepatosplenomegaly, and pneumonia [14,15]. In our study, we have observed CMV transmission in only 1 of 47 (2.5%) preterm infants, who had clinical symptoms related to CMV infection (sepsis-like symptoms and hepatosplenomegaly), although the viral load and detection rate (70.2%) of CMV DNA in the breast milk were high. The discrepancy between our study and other reports may not be associated with the studied population, since the mean gestational age and birth weight were similar (29 vs 29 weeks; 1158 vs 1100 g, respectively), rather with the difference in breast milk storage. In fact, before feeding, the breast milk has been kept and preserved at -20°C for 72 hours long care of in our lactarium. Hamprecht et al. previously have reported that 25% of preterm infants had acquired CMV infections, after feeding with raw breast milk refrigerated at 4 to 10°C for a maximum of 12 hours [10-12]. CMV infections have not been observed in CMV seronegative preterm infants, fed with banked human milk which was either pasteurized or frozen [14-17]. However, while detection of CMV after heating (72°C for 10 seconds) tested negative, freezing can reduce only partially CMV infectivity, especially when the virus load was high [18]. This may explain the occurrence of the transmission of CMV in one infant.

In conclusion, our study confirms that CMV transmission through breast milk can cause a severe clinical course in preterm infants but freezing could highly decrease the transmission rate. Because breastfeeding is healthy and wide spread, and the number of preterm infants is increasing in the most developed countries, a new procedure for gentle virus inactivation of seropositive breast milk is being assessed in our laboratory, to prevent CMV transmission to extremely preterm infants in the future [19,20].

## Abbrevations

CMV: CitoMegaloVirus; LBW: Low Birth Weight; NGT: NasoGastric Tube; NICU: Neonatology Intensive Care Unit; PCR: Polymerase Chain Reaction; RT-PCR: Real Time PCR;

## Competing interests

The authors declare that they have no competing interests.

## Authors' contributions

MC carried out alignment and drafted the manuscript and performed the statistical analysis. PB conceived of the study, and participated in its design and coordination. AS participated in the design of the study. EC carried out CMV culture.

RC carried out the immunoassays and PCR. MJR participated in the design of the study. AF participated in the sequence alignment.

All authors read and approved the final manuscript

## References

[B1] Stagno S Cloud GA Working parents: the impact of day care and breast-feeding on cytomegalovirus infections in offspring Proc Natl Acad Sci USA 1994 91 2384 9 10.1073/pnas.91.7.2384 8146127 PMC43376

[B2] Hamprecht K Goelz R Maschmann J Breast milk and cytomegalovirus infection in preterm infants Early Human Development 2005 81 989 96 10.1016/j.earlhumdev.2005.10.009 16278059

[B3] Jim WT Shu CH Chiu NC Kao HA Hung HY Chang JH Peng CC Hsieh WS Liu KC Huang FY Transmission of cytomegalovirus from mothers to preterm infants by breast milk Pediatr Infect Dis J 2004 23 9 848 51 10.1097/01.inf.0000137571.35541.55 15361725

[B4] Maschmann J Hamprecht K Dietz K Jahn G Speer CP Cytomegalovirus infection of extremely low-birth weight infants via breast milk Clin Infect Dis 2001 33 1998 2003 10.1086/324345 11712092

[B5] Nigro G Scholz H Bartmann U Ganciclovir therapy for symptomatic congenital cytomegalovirus infection in infants: A two-regimen experience The Journal of Paedriatrics 1994 124 2 318 322 10.1016/s0022-3476(94)70327-2 8301446

[B6] Boeckh M Guy B Quantitation of Cytomegalovirus: Methodologic Aspects and Clinical Applications Clinical Microbiology Reviews 1998 3 11 533 54 10.1128/cmr.11.3.533 PMC88895 9665982

[B7] Drew WL Laboratory diagnosis of cytomegalovirus infection and disease in immunocompromised patients Curr Opin Infect Dis 2007 20 4 408 11 10.1097/QCO.0b013e32821f6010 17609601

[B8] Sensini A Cytomegalovirus infection in pregnant woman fetus and newborn: evaluation of different diagnostic procedures13th European Congress of Clinical Microbiology and Infection Diseases, Glasgow, Scotland 2003 1191 abstract

[B9] Forsgren M Cytomegalovirus in breast milk: reassessment of pasteurization and freezethawing Pediatr Res 2004 56 526 8 10.1203/01.PDR.0000143155.84802.A3 15388851

[B10] Hamprecht K Maschmann J Vochem M Dietz K Speer CP Jahn G Epidemiology of transmission cytomegalovirus from mother to preterm infants by breast feeding Lancet 2001 357 513 8 10.1016/S0140-6736(00)04043-5 11229670

[B11] Hamprecht K Witzel S Maschmann J Speer CP Jahn G Transmission of cytomegalovirus infection through breast milk in term and preterm infants: the role of cell free milk whey and milk cells Adv Exp Med Biol 2000 478 231 9 full_text 11065076

[B12] Vochem M Hamprecht K Jahn G Speer CP Transmission of cytomegalovirus to preterm infants through breast milk Pediatr Infect Dis J 1998 17 53 8 10.1097/00006454-199801000-00012 9469396

[B13] Dworsky M Yow M Stagno S Pass RF Alford C Cytomegalovirus infection of breast milk and transmission in infancy Pediatrics 1983 72 295 9 6310479

[B14] Yeager AS Palmubo PE Malachowski N Ariagno RL Stevenson DK Sequela of maternally derived cytomegalovirus infections in premature infants J Pediatr 1983 102 918 22 10.1016/S0022-3476(83)80025-0 6304275

[B15] Kumar ML Nankervis A Cooper AR Gold E Postnatally acquired cytomegalovirus infections in infants of CMV-excreting mothers J Pediatr 1984 104 669 73 10.1016/S0022-3476(84)80941-5 6325653

[B16] Yeager AS Grumet FC Hafleigh EB Arvin AM Bradley JS Prober CG Prevention of transfusion-acquired cytomegalovirus infections in newborn infants J Pediatr 1981 98 281 7 10.1016/S0022-3476(81)80662-2 6257877

[B17] Dworsky M Stagno S Pass RF Cassady D Alford C Persistant of cytomegalovirus in human milk after storage J Pediatr 1982 101 440 3 10.1016/S0022-3476(82)80081-4 6286914

[B18] Maschen J Speer CP Jahn G Hamprecht K Inactivation of cytomegalovirus (CMV) in breast milk European Society for Pediatric Research. Pediatr Res 1999 45 6 924 9

[B19] WHO Collaborative Study Team on the Role of Breastfeeding on the Prevention of Infant Mortality Effect of breastfeeding on infant and child mortality due to infectious diseases in less developed countries: a pooled analysis Lancet 2000 355 451 5 10841125

[B20] Hamprecht K Maschmann J Jahn G Poets CF Goelz R Cytomegalovirus transmission to preterm infants during lactation J ClinVirol 2008 41 3 198 205 10.1016/j.jcv.2007.12.005 18243784

